# Significant Changes in Metabolic Profiles after Intervention with Selenium and Coenzyme Q10 in an Elderly Population

**DOI:** 10.3390/biom9100553

**Published:** 2019-09-30

**Authors:** Urban Alehagen, Peter Johansson, Jan Aaseth, Jan Alexander, Izabella Surowiec, Katrin Lundstedt-Enkel, Torbjörn Lundstedt

**Affiliations:** 1 Division of Cardiovascular Medicine, Department of Medical and Health Sciences, Linköping University, SE-581 85 Linköping, Sweden; 2 Department of Social and Welfare Studies, Linköping University, SE-581 83 Linköping, Sweden; peter.b.johansson@liu.se; 3 Department of Internal Medicine, Linköping University, SE-581 83 Linköping, Sweden; 4 Department of Medical and Health Sciences, Linköping University, SE-581 83 Linköping, Sweden; 5 Research Department, Innlandet Hospital Trust, N-2380 Brumunddal, Norway; jaol-aas@online.no; 6 Norwegian Institute of Public Health, N-0403 Oslo, Norway; Jan.Alexander@fhi.no; 7 AcureOmics AB, Tvistevägen 48, SE-907 36 Umeå, Sweden; izabella.surowiec@acureomics.com (I.S.); katrin.lundstedt-enkel@telia.com (K.L.-E.); torbjorn.Lundstedt@acureomics.com (T.L.)

**Keywords:** selenium, coenzyme Q10, elderly, metabolic profiling

## Abstract

Selenium and coenzyme Q_10_ (SeQ_10_) are important for normal cellular function. Low selenium intake leads to increased cardiovascular mortality. Intervention with these substances with healthy elderly persons over a period of four years in a double-blind, randomised placebo-controlled prospective study showed reduced cardiovascular mortality, increased cardiac function, and a lower level of NT-proBNP. Therefore, we wanted to evaluate changes in biochemical pathways as a result of the intervention with SeQ_10_ using metabolic profiling. From a population of 443 healthy elderly individuals that were given 200 µg selenium and 200 mg coenzyme Q_10_, or placebo daily for four years, we selected nine males on active intervention and nine males on placebo for metabolic profiling in the main study. To confirm the results, two validation studies (study 1 *n* = 60 males, study 2 *n* = 37 males) were conducted. Principal component analyses were used on clinical and demographic data to select representative sets of samples for analysis and to divide the samples into batches for analysis. Gas chromatography time-of-flight mass spectrometry-based metabolomics was applied. The metabolite data were evaluated using univariate and multivariate approaches, mainly T-tests and orthogonal projections to latent structures (OPLS) analyses. Out of 95 identified metabolites, 19 were significantly decreased due to the intervention after 18 months of intervention. Significant changes could be seen in the pentose phosphate, the mevalonate, the beta-oxidation and the xanthine oxidase pathways. The intervention also resulted in changes in the urea cycle, and increases in the levels of the precursors to neurotransmitters of the brain. This adds information to previous published results reporting decreased oxidative stress and inflammation. This is the first-time metabolic profiling has been applied to elucidate the mechanisms behind an intervention with SeQ_10_. The study is small and should be regarded as hypothesis-generating; however, the results are interesting and, therefore, further research in the area is needed. This study was registered at Clinicaltrials.gov, with the identifier NCT01443780.

## 1. Introduction

Selenium (Se) is a trace element that is essential for all living cells within the human body. There are 25 genes encoding for selenoproteins in humans [1]. Several of the selenoproteins have important roles in anti-oxidative stress defence, and in immune surveillance [2,3]. Some of the most important selenoproteins are the glutathione peroxidases, thioredoxin reductase 1, selenoprotein P, and iodothyronine deiodinases [4]. There is a relation between the Se content in soil, food grown on these soils and dietary Se intake, where both high and low intakes have been reported [5,6]. In Europe there is in general a low concentration of Se in the soil [7,8,9,10,11] and, hence, a low intake, estimated at about 40 µg Se/day [1]. The daily intake of selenium needed for the optimal expression of selenoprotein P in plasma has been debated. Xia et al. demonstrated that an intake of at least 75 µg/day, to achieve a plasma concentration of Se above 90 µg/L was required in normal weight Caucasians [12], whereas Hurst et al. reported that an intake of at least 105 µg/day was required [13]. In Sweden, reports have shown an intake of selenium below the recommended levels [14,15], which appears to increase the cardiovascular risk [16]. The soils in North America have a significantly higher content of selenium than in Europe, with reported serum selenium levels generally above 120 μg/L in the US population [17,18]. In a population of elderly healthy Swedish persons with a mean serum selenium concentration of 67 μg/L, we showed that those with low selenium concentration had a higher cardiovascular risk compared to those with higher selenium concentration, thus confirming previous reports in the literature [16,19]. 

There is an important relationship between Se and ubiquinone (coenzyme Q_10_, hereafter abbreviated Q_10_) in living cells. In order to synthesise selenocysteine-containing enzymes a functional mevalonate pathway is required, and Q_10_ is a product in this pathway [20]. Additionally, the selenoenzyme TrxR may reduce Q_10_ to its active form_,_ ubiquinol; hence, the presence of an adequate concentration of selenium is required in the cell for optimal Q_10_ function. 

In addition to being a strong antioxidant and essential for all living cells, Q_10_ is of particular importance for the crucial ATP-generating steps within the mitochondrial respiratory chain and in oxidative phosphorylation mediated through the pentose phosphate pathway [21]. The endogenous production of Q_10_ decreases with increasing age, and in the myocard, by the age of 80 years it is reduced to half the level that exists at 20 years [22]. Furthermore, the need for Q_10_ is even greater during diseases [23,24]. 

In 2013, we presented the results of four years of intervention using Se and Q_10_ combined (SeQ_10_) as a dietary supplement in an elderly healthy population in a randomised prospective double-blind, placebo-controlled study [25]. The main result of the intervention was a significant reduction of cardiovascular mortality by more than 50%, better cardiac function as demonstrated on echocardiography, and a lower plasma concentration of the natriuretic peptide N-terminal fragment of proBNP (NT-proBNP) indicating less myocardial wall tension [25]. Due to the striking positive clinical results, in an effort to shed light on possible mechanisms behind the clinical effects, we used a metabolic profiling approach to pinpoint changes in metabolites and metabolic pathways following SeQ_10_ administration. 

Studies of metabolic patterns, by analysing body fluids or sampled tissue, have evolved into an important routine in several fields of the clinical and pharmacological discipline [26]. Non-targeted metabolite profiling—known as metabolomics—provides a functional molecular picture of the biochemistry connecting both genomics and proteomics to a physiological state, and it also incorporates environmental interactions. Metabolomics is, thus, a mapping of the actual state of the phenotype.

The metabolomic approach in characterising biological systems is frequently applied in several situations; (1) In the diagnostic process, i.e., where the metabolic profiles of healthy subjects, and subjects with a disease are used for classification of new cases. (2) In the identification of perturbed metabolic pathways, i.e., the detection of biomarkers and connecting them to, e.g., disease pathogenesis, and (3) as a tool in disease follow-up and treatment, i.e., following changes of metabolites after changes in lifestyle and/or food and/or pharmacological intervention [27,28,29,30]. 

In the present work, we used metabolomics to identify the impacts of SeQ_10_ intervention on the plasma metabolome. As previous results from this intervention study indicated that males and females differed strongly in cardiovascular mortality, and as we could not exclude a hormonal influence, we decided to include only males in this study in order to have a more homogenous group to analyse.

## 2. Results

The baseline characteristics of the total male study population are presented in Table 1.

As can be seen, all variables were balanced between the two groups included in this evaluation, with the exception that among those receiving a placebo there were more participants in the NYHA functional group II and fewer in group I.

### 2.1. Main Study 

**Figure 1:**
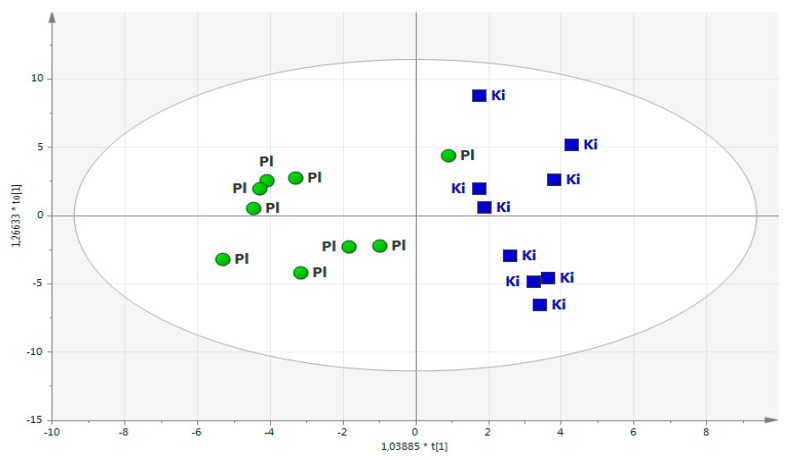
OPLS-DA score plot based on each individual’s metabolic profile. (R^2^X = 0.19, R^2^Y = 0.57, Q^2^Y = 0.18). Note: Round circles indicate the men that received the placebo (Pl, green; *n* = 9), and the squares are men treated with SeQ_10_ (Ki, blue; *n* = 9). Note: The metabolite pattern was determined in plasma at the time point of 18 months (T_18_). Note: One of the individuals receiving placebo took SeQ_10_ by self-administration.

The active treatment group was evaluated against the placebo group using the OPLS-DA model assessment at 18 months (T18) where 95 metabolites analysed in plasma were included. This evaluation demonstrated significant differences between the two groups by using seven-fold cross-validation (the OPLS-DA model with one significant component that explained 57% of the variance in Y, R^2^X = 0.19, R^2^(cum) = 0.57 and Q^2^(cum) = 0.18) (Figure 1). 

One of the samples from the placebo group had a position in the plot that indicated scores that deviated from those of the rest of the placebo group and, thus, negatively influenced the power of the OPLS-DA model used. After checking with this individual, it was revealed that despite being a participant of the placebo group, the person with sample no. 186 supplemented himself with selenium on his own initiative, which the model including each individual’s metabolite pattern in plasma, could detect and demonstrate (Figure 1). 

**Figure 2:**
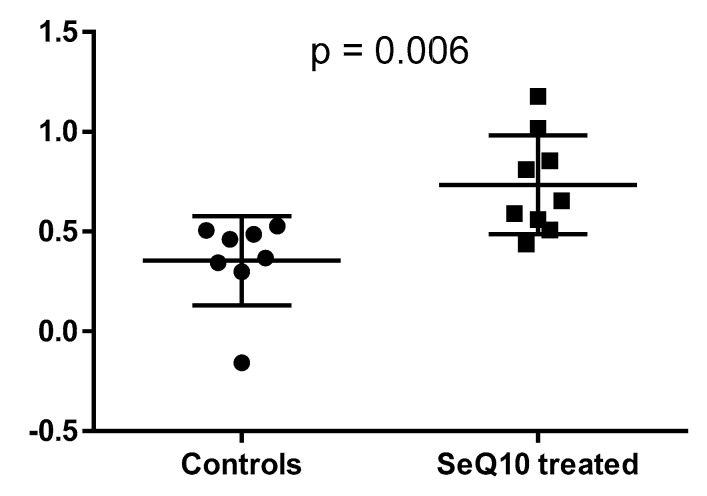
Predicted cross-validated Y values (from the OPLS-DA model). Note: Actual values for controls are denoted 0 and for SeQ_10_-treated are denoted 1, showing that the two groups’ metabolic patterns are significantly different (*p* = 0.006).

This individual was thus excluded from all further modelling, resulting in the final OPLS-DA model with one predictive and one orthogonal component including 17 individuals (R^2^X = 0.13, R^2^(cum) = 0.91, Q^2^(cum) = 0.41). The difference in the whole metabolome between the two studied groups was evident when comparing predicted cross-validated Y values for samples from both groups (*p* < 0.01) (Figure 2). 

Using the jack-knifing method in the multivariate analyses and applying a 95% confidence interval, the results showed that 19 metabolites out of the 95 identified and modelled metabolites were significantly decreased in the SeQ_10_-treated men. A further examination applying a T-test analysis, showed that 13 of these 19 metabolites were significantly lower in the SeQ_10_ -treated men when using univariate statistics (Table 2). 

**Figure 3:**
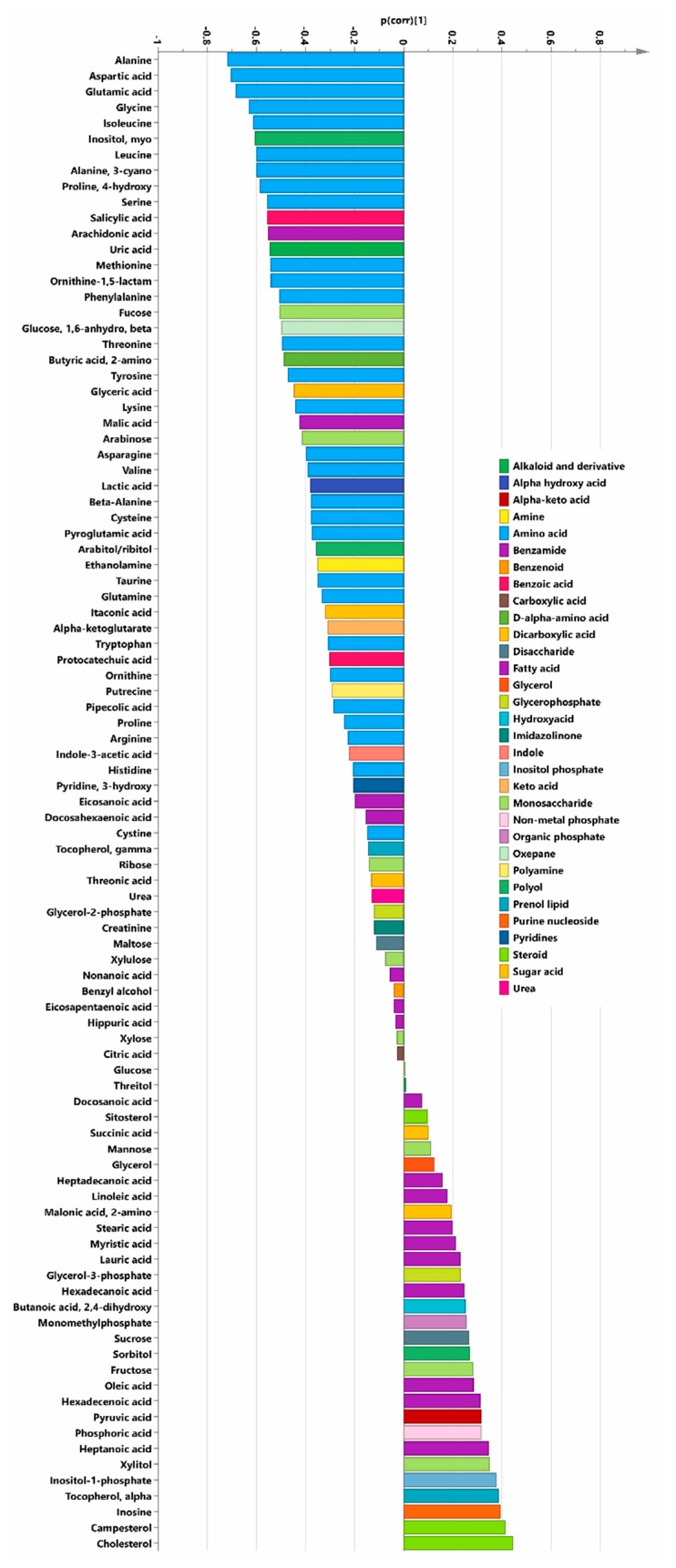
P(corr) loading vector from the OPLS-DA model with the 95 metabolites identified in plasma of men (*n* = 9) receiving a daily addition of SeQ_10_ for 18 months as compared with placebo-treated men (controls; *n* = 8). Note: Bars pointing to the left from mean (0) indicate metabolites with lower levels in SeQ_10_-treated men. Amino acids are marked in light blue.

No metabolites were significantly higher in the SeQ_10_ men as compared with the controls. The p(corr) loading plot from the OPLS-DA model indicated that the difference could largely be attributed to changes in amino acids, which were at lower levels in the SeQ_10_-treated group in comparison with the placebo group (Figure 3). 

However, a decrease could also be seen in the polyunsaturated fatty acids arachidonic acid, eicosapenatenoic and docosahexaenoic acid, and an increase in the saturated fatty acids (hexadecanoic acid, myristic and stearic acid) and monounsaturated fatty acids hexadecenoic acid and oleic acid.

### 2.2. Projection of Deceased Participants

Two individuals that participated in the project for at least 18 months, but later died from cardiovascular disease, delivered samples at T0 and T18. The metabolic profiles from these individuals were projected into the principal component analysis (PCA) models to investigate whether the T18 blood samples from these individuals deviated from the other T18 samples. In the score plots (not shown) it could be seen that the metabolic patterns of the samples from the deceased participants did not deviate from the surviving participants. It was, therefore, not possible to use the existing models to predict the fate of individual participants after T18.

### 2.3. Validation Studies 

Both univariate analyses and multivariate modelling were carried out in each batch separately. The results are summarised in Table 3 and Table 4 where the T-tests with corresponding variables’ importance to the projection (VIP) values from the OPLS-DA models are presented, and the values of the combined loading vectors obtained are presented in Table 5. In that table, 62 out of 107 (57.9%) evaluated metabolites showed congruence in change, or no change between the main study and at least one of the validation studies.

### 2.4. Validation Studies 1 and 2

Whereas no metabolites by univariate analysis were significantly changed in any of the studies, comparison of metabolic profiles in relation to the SeQ_10_ administration supports the results from the main study at T18. There is also consistency between the two validation studies, where 62% of the metabolites detected in at least two studies showed the same changes (higher or lower metabolite levels) in SeQ_10_-treated individuals as compared with those receiving a placebo. The differences between the groups were largely attributed to amino acids that were at lower levels in the actively treated group compared with the placebo group (Table 3). 

## 3. Discussion

Our group has recently presented clinical data showing reduced cardiovascular mortality, increased cardiac function according to echocardiographic evaluation, and decreased concentration of the cardiac biomarker NT-proBNP, as a result of intervention with SeQ_10_ as a dietary supplement given to an elderly healthy population over four years. To acquire knowledge of the possible biochemical mechanisms behind these clinical results, we have applied metabolic profiling to investigate possible metabolic changes due to the intervention. 

We have shown an effect on the metabolome in the SeQ_10_-treated men after 18 months when compared with those receiving the placebo (Figure 1, Figure 2 and Figure 3, Table 2 and Table 3). 

For a majority of the changes observed in the main study (57.9%), the same changes could be seen in one or both validation studies, supporting the validity of the results from the main study. Together, the changes in the metabolite patterns after SeQ_10_ treatment could be attributed to biochemical pathways discussed further in the following paragraphs. 

An interesting example of the effect of the intervention could be seen in the level of 1,5-anhydro-D-glucitol, a monosaccharide that is found in many foods. This substance is reabsorbed by the proximal renal tubule in competition with glucose. A high blood concentration of 1,5-anhydro-D-glucito is indicative of a low level of glucose in primary urine, and is therefore regarded as positive [31]. It has also been reported in the literature that low levels of 15-anhydro-D-glycitol are associated with increased cardiovascular mortality or increased coronary artery disease risk [32], even in cases without signs of diabetes [33]. In our study we reported a higher concentration of the substance in those receiving SeQ_10_, compared with those on placebo, indicating a change in the metabolomic profile that could be a clinically important effect of the intervention. 

### 3.1. Biochemical Pathways Altered by the Intervention with SeQ_10_

(A) The pentose phosphate pathway, which is an alternative metabolic pathway to that of glycolysis with generation of, e.g., NADPH and 5-carbon sugars (pentoses), was enhanced. In those receiving SeQ_10,_ a decrease in 1,6-anhydro-β-D-glucose, as well as an increase in glycerol-3-phosphate, fructose and inositol-1-phosphate could be seen. This strongly indicates that the equilibria are shifted towards the generation of reducing equivalents in the form of NADPH, i.e., further providing selenoproteins, e.g., thioredoxin reductase, glutathione peroxidases and selenop, that may scavenge reactive oxygen species (ROS) within the cell. Previously, increased oxidative stress with ROS formation has been discussed as one of the main mechanisms in the development of heart disease [34]; therefore, intervention to decrease oxidative stress is of great interest [35]. Our group have previously reported signs of less oxidative stress as a result of the SeQ_10_ intervention [36].

(B) The mevalonate pathway, in which squalene is an important precursor in the biosynthesis of cholesterol, heme, vitamin K, coenzyme Q and all steroid hormones [37], was influenced by the intervention with selenium and coenzyme Q10. We observed that several phytosterols e.g., campesterol, and sitosterol showed higher levels in the SeQ_10_-treated participants than in those receiving the placebo. Usually, phytosterols are exogenous compounds produced by plants and found in the diet. Some phytosterols may be added to food (usually margarine), and they compete with cholesterol as regards intestinal absorption and can thereby lower LDL cholesterol [38]. It has been reported that phytosterols reduce LDL levels and increase HDL in the blood and, thus, have a potential place in the treatment of hyperlipidaemia [39,40,41]. A potential source of the phytosterols in the present study could be the yeast in the tablets containing yeast selenium. One might also speculate that the intervention modified the gastro-intestinal flora, resulting in increased intestinal release of phytosterols from the food matrix or uptake of such compounds [42].

(C) The beta-oxidation pathway is increased by the treatment with SeQ_10_. This is supported by the decrease of unsaturated fatty acids with an even number of double bonds and the increased levels of monounsaturated fatty acids (Figure 3). Thus, fatty acids with multiple double bonds decrease, whereas less unsaturated fatty acids increase as a result of the intervention. In combination with the cardio-protective steroids produced in the mevalonate pathway that could be demonstrated in the analyses in the present study, the altered fatty acid composition indicates that SeQ_10_ should have a positive effect on individuals with metabolic syndrome and/or type 2 diabetes [43]. 

(D) The xanthine oxidase pathway, which is a metabolic pathway for uric acid formation, is down-regulated as the levels of uric acid in plasma are decreased in SeQ_10_ -treated men. Uric acid is a major antioxidant in a hydrophilic environment. Reduced synthesis might indicate a reduced need for antioxidants because ROS are taken care of by selenoproteins and/or the reduced form of Q_10_ in the supplemented group. This supports our previous results that SeQ_10_ appeared to have an anti-inflammatory effect [44]. From a clinical point of view, reports indicating an important association between inflammation and vascular disease can be found in the literature. Therefore, this aspect is important [45,46]. An anti-oxidative effect caused by the intervention with SeQ_10_ and shown by the decrease in uric acid levels is also supported by previously presented findings in the same study population on the concentrations of copeptin and the midregional segment of proadrenomedullin (MR-proADM) as markers for anti-oxidative activity [36]. 

(E) An interesting observation is that an influence on the urea cycle could be seen, with decreased levels of non-essential amino acids as well as creatinine, but without increased levels of the corresponding ketoacids. It could be inferred that the non-essential amino acids were part of the production of proteins. This suggests that the treatment with SeQ_10_ favours an anabolic process instead of an oxidative process with catabolism. 

Decreased levels of the essential amino acids could also be seen in those who were supplemented with SeQ_10_, compared with those on placebo. As it is reported in the literature that a high concentration of some essential, branched-chain amino acids (leucine, isoleucine and valine) is associated with diabetes mellitus [47,48], and also with oxidative stress and inflammation [49], decreased levels could be interpreted as positive.

### 3.2. General Effects of SeQ_10_ Treatment

In addition to the changes discussed above we also observed that the levels of the precursors to neurotransmitters of the brain, i.e., the aromatic amino acids, were decreased in those receiving SeQ_10_. This might explain the observed reduction in sense of fatigue in the intervention group reported previously [50] The same has also been reported for Q_10_ given in monotherapy [51,52].

An interesting observation is that the participants treated with SeQ_10_ had changes in their metabolome that were opposite to changes in patients suffering from severe systemic lupus erythematosus (SLE) [53]. Therefore, patients with severe SLE might also benefit from intervention with SeQ_10_, as this could normalise their pathological metabolic profile.

At T18 months we also observed that the SeQ_10_-treated men had lower levels of salicylic acid in their plasma, which could imply less consumption of salicylic acid-containing painkillers than the placebo group. However, we do not have any anamnestic data to support this interesting observation, even though there are reports in the literature indicating such an effect by coenzyme Q10 [54,55,56].

Another observation was decreased levels of the essential amino acid methionine in the SeQ_10_-treated group. Methionine is the main precursor for cysteine and glutathione and a central molecule in one-carbon metabolism as a precursor for S-adenosylmethionine (SAM), the principal donor of methyl groups. In mice, selenium modulates one-carbon metabolism and increases hepatic methylation capacity, resulting in increased global DNA methylation and affecting lipogenesis [43,57]. The DNA methylation regulating gene expression is also related to ageing [58]. In a previous publication our group presented data indicating less fibrosis in seven out of eight evaluated biomarkers for fibrosis in the population receiving SeQ_10_ compared with those on placebo. This could indicate a possible anti-ageing effect on the cardiovascular system, as the ageing process is highly associated with increased fibrosis of the cardiovascular system [59]. Increased expression and synthesis of selenoprotein P following selenium supplementation requires SAM-dependent protein methylation [57]. 

Taken together, the presented changes in metabolic profiles as seen in the present evaluation could provide important information to support the positive clinical results that have been demonstrated in the previous publications. Therefore, for those with insufficient selenium and coenzyme Q_10_ intake, the metabolic profiling results could strengthen the argument for supplementation.

## 4. Material 

### 4.1. Aim 

The aim of the present work was to investigate changes in biochemical pathways after intervention with SeQ_10_ to a healthy elderly male community-living population by metabolic profiling of plasma metabolites.

### 4.2. Design

A double-blind randomised placebo-controlled study where intervention with a dietary supplement of Se and Q_10_ combined (SeQ_10_) in an elderly healthy rural population of 443 individuals in the age range of 70–88 years has been previously reported [25,60]. The first participant was included in January 2003, and the last participant concluded the study in February 2010. The participants received the intervention for 48 months during which time they were re-examined every six months, and throughout the study, all mortality was registered. During the study 221 individuals received active supplementation of 200 μg/day organic Se (SelenoPrecise^®^, PharmaNord, Denmark), plus 200 mg/day of Q_10_ (Bio-Quinon^®^, PharmaNord, Denmark), and 222 individuals received a placebo. At inclusion, all participants underwent clinical examination, a new patient record was obtained, the New York Heart Association (NYHA) functional class was assessed (this classification grades how a patient with heart disease experiences symptoms of tiredness, breathlessness or chest pain; it is graded from I–IV, where IV is symptoms already at rest), an ECG and a Doppler-echocardiography were performed. Informed consent was obtained from each patient. The study was approved by the Regional Ethical Committee in Linköping (Forskningsetikkommmitten, Hälsouniversitetet, SE-581 85 Linköping, Sweden; no. D03-176), and it conforms to the ethical guidelines of the 1975 Declaration of Helsinki. The Medical Product Agency declined to review the study protocol since the study was not considered a trial of a medication for a certain disease, but rather one of food supplement commodities that are commercially available. The study was registered at clinicaltrials.gov. (NCT01443780).

## 5. Methods

### 5.1. Echocardiography 

Doppler-echocardiography examinations were performed with the participants in the left lateral position. The ejection fraction (EF) readings were categorised into four classes with interclass limits placed at 30%, 40% and 50% [61,62]. Normal systolic function was defined as EF ≥ 50%, while severely impaired systolic function was defined as EF *<* 30%. 

### 5.2. Blood Samples

Blood samples were collected while the participants were resting in a supine position. Pre-chilled, EDTA vials (Terumo EDTA K-3) were used. The vials were placed on ice and centrifuged for 10 min at 3000× *g*, +4 °C, and the plasma was immediately transferred to storage at −70 °C until further analysis. No sample was thawed more than once. 

### 5.3. Sample Selection

The available biobank contained samples from 443 individuals, including both males and females, and the male population was chosen. From those, the individuals who delivered blood samples at one (T0), two (T0 + T18 months), or three time points (T0 + T18 months + T48 months) were chosen after taking into account clinical and demographic variables in order to get balanced groups. The samples were divided into a main study as well as two validation studies. In each of the validation studies the samples were divided into analytical batches run on separate days. Each batch contained a balanced mix of treatment and placebo samples, with all time point samples from an individual included in the same batch. As explained below, samples selected for analysis were representative of the whole study and covered the whole expected intervention period, the same was true for batch separation where each batch itself contained representative information found in the whole cohort [63,64]. Therefore, the individuals included in the study were selected to meet those criteria.

### 5.4. Main Study

In the participant database, clinical and demographic data were used for the selection of participants. From the original dataset, a selection was made by use of multivariate design (MVD), which, in turn, was based on a combination of an experimental design and multivariate data analysis (MVDA) [63,64]. By using MVD we ensured that the selected subjects together spanned as large an area of the available properties domain as possible, thus including as large a natural variation as possible, and at the same time this ensured that the properties’ domain in the placebo group covered the same domain as the group receiving SeQ_10_. Principal component analysis of the clinical and demographic information that was collected on the inclusion date of each participant was applied. A total of 182 variables representing all available demographic and clinical data were used in the PCA model. Based on two separate overview models of the SeQ_10_ and the placebo-treated men, a representative selection for an initial metabolomic study was obtained. From the PCA score plot (which plotted the subjects in the first two principal components) 10 individuals were selected from each group (two from each of the four quadrants plus two from the centre, thus spanning the whole properties domain). In addition to these 20 participants, seven others (two SeQ_10_ and five placebo) were selected representing the participants that had participated up until T18 but had later died of cardiovascular disease (CVD). Those who died a cardiovascular death (*n* = 2) during the intervention time were not included in the main analyses but were evaluated separately. 

When retrieving the samples from the freezer it was noticed that one of the placebo samples was contaminated with hepatitis and this was therefore excluded from analysis. During sample preparation, one of the SeQ_10_ samples did not derivatise so no metabolite analysis could be performed.

All derivatised samples were analysed by GC-MS, and 315 metabolites were detected. Using metabolite databases, 95 of these were identified and were used in the modelling of metabolite profiles.

Ultimately, we included nine male SeQ_10_ and nine placebo participants (T18) in the main study, and we treated the samples from the individuals that died from CVD as a separate group.

### 5.5. Validation Studies

To confirm the results from the main study, it was decided to perform two validation studies; “Validation study 1” and “Validation study 2”, each made up of several batches. Each batch contained a balanced mix of SeQ_10_ and placebo samples, selected according to the MVD approach (as described for the main study). 

#### 5.5.1. Validation Study 1

Metabolic profiles from male individuals were investigated. A total of 60 male individuals (31 SeQ_10_, 29 placebo) were included, all having three samples each (T0, T18 and T48). One individual (SeQ_10_) from the first study with two time points was included as well. All samples belonging to Validation study 1 were divided into five analytical batches (subsets).

#### 5.5.2. Validation Study 2

In validation study 2, samples from 37 men (23 SeQ_10_, 14 placebo) were included. 

### 5.6. Metabolomic Profiling

#### 5.6.1. Metabolite Analysis (Extraction, Quantification and Identification)

Plasma samples were extracted, derivatised, and analysed using GC–TOF–MS as previously described [65]. Masses were acquired in the mass range 50–800 mass/charge numbers of ions (m/z) at a rate of 30 spectra/s. The acceleration voltage was turned on after a solvent delay of 150 s. An alkane series (C10–C32) was run together with all samples. Non-processed MS files from GC/TOFMS analysis were exported in NetCDF format to MATLAB software 7.3 (Mathworks, Natick, MA, USA), where all data pre-treatment procedures, such as baseline correction, chromatogram alignment, time-window setting and multivariate curve resolution were performed using custom scripts [66]. 

For the identification of metabolites, The National Institute of Standards and Technology (NIST) MS Search software (version 2.0, Gaithersburg, MD, USA) was used. With this we compared the mass spectra of all detected compounds with spectra in the NIST library, the in-house mass spectra library established by Umeå Plant Science Centre and Swedish Metabolomics Centre and the mass spectra library maintained by the Max Planck Institute, Golm, Germany (http://csbdb.mpimp-golm.mpg.de/csbdb/gmd/gmd.html). A retention index comparison was performed, with a retention index deviation < ±10 (in addition to a high spectral match, above 800). 

#### 5.6.2. Statistical Analysis

##### Univariate Statistics

Univariate analysis was performed on each metabolite separately, with *p* values calculated by performing two-sample, unequal variance, two-tailed *t*-tests.

##### Multivariate Data Analysis 

Metabolite levels reflect biochemical processes, and as few processes in an organism are truly independent, metabolite levels are also highly correlated. The MVDA approaches are best suited to analyses of highly correlated data and allow the identification of the following: small changes in the variable patterns between different groups; internal relations/correlations between samples and between metabolites; and how samples and metabolites are connected to each other. These differences can be missed by using a univariate approach alone [67,68,69].

##### OPLS-DA Models

The orthogonal partial least-squares discriminant analysis (OPLS-DA) models were calculated between the two intervention groups (SeQ_10_ and placebo) to identify the metabolites that showed different levels in these groups.

##### Data Normalisation

Data were normalised using 11 internal standards (eluting over the whole chromatographic time range). To obtain accurate peak areas for the internal standards, two unique masses for each compound were specified and the samples were reprocessed using an in-house MATLAB-based script. Principal component analyses were performed on the peak areas of internal standards with Unit Variance Normalisation and the first component (t_1_) score value for each sample was used to normalise the resolved data. This was done by dividing the peak areas in each sample by the corresponding score value [70]. 

All multivariate modelling was performed using the software SIMCA version 14 (Umetrics, Umeå, Sweden). The PCA approach was used for a metabolomics data overview [71], and the OPLS-DA approach was used to elucidate the differences concerning metabolomics between the two groups of subjects (SeQ_10_ and placebo) [72,73]. Unit Variance scaling was used for both types of models [74]. The significant differences between the SeQ_10_ group and the placebo-treated individuals were identified by using confidence intervals calculation by use of jack-knifing [75]. A potential model significance was found by means of cross-validation [75]. Furthermore, cross-validated predicted Y values from the cross-validation procedure were used to visualise the ability of the OPLS-DA model to separate individuals receiving SeQ_10_ from those receiving a placebo.

The VIP values were calculated (SIMCA v.14) as VIP scores. This was done in order to estimate the importance of each variable in the projection used in the OPLS-DA model. A variable with a VIP score of about 0.8 or greater was considered significant in the model.

In order to obtain an overview model of the different batches, the p(corr)-loadings for each of the SeQ_10_ treated vs. placebo OPLS-DA models were combined into an X-matrix which was used to create a PCA model where no scaling was applied. The p [1] loading profile from this PCA model was used as a combined metabolic profile representing the metabolic effects of the SeQ_10_ intake in validation studies 1 and 2, and compared with the p(corr) vector from the main study.

Small differences in the number of significant compounds when detected by univariate and multivariate approaches were expected, since both approaches are based on different testing principles; the former (*t*-tests) is a statistical hypothesis test, whereas the multivariate testing, using jack-knifing, is a resampling technique applied to the multivariate space. These differences were, thus, included in the evaluation of the results.

## 6. Conclusions

There was a clear difference in the metabolic profile in plasma from men receiving SeQ_10_ as compared with men receiving placebo. The major differences can be seen in the pentose phosphate pathway, in the mevalonate pathway, and in the beta-oxidation pathway. An interesting observation was downregulation of plasma methionine, indicating increased methylating activity, as also previously observed experimentally. It can also be reported that xanthine oxidase was down-regulated. Moreover, there were effects on the urea cycle where the non-essential amino acids were down-regulated. All these effects, together with signs of less inflammation and less oxidative stress observed in the previous studies of this group, indicate a positive health effect by the supplementation with selenium and coenzyme Q_10_ compared with those on placebo.

However, as this is the first intervention with the SeQ_10_ combination in an elderly population low in Se, the results should be regarded as hypothesis-generating, and more research is needed to confirm the positive health impact of the SeQ_10_ combination in elderly persons. 

## Figures and Tables

**Table 1:** Baseline characteristics of the male study population receiving an intervention of dietary supplementation of selenium and coenzyme Q_10_ combined over four years.

	Active	Placebo	*p* Value
N	55	44	
Age (years)	76.1 (2.9)	76.3 (2.9)	
**History**			
Smokers (present) n (%)	5 (7.3)	6 (13.6)	0.47
Diabetes n (%)	11 (20.0)	8 (18.2)	0.82
Hypertension n (%)	34 (61.8)	31 (70.5)	0.91
IHD n (%)	12 (21.8)	10 (22.7)	0.91
NYHA class I n (%)	37 (67.3)	22 (50.0)	0.08
NYHA class II n (%)	12 (21.8)	18 (40.9)	0.04
NYHA class III n (%)	6 (10.9)	4 (9.1)	0.77
NYHA class IV n (%)	0	0	
Unclassified NYHA n (%)	0	0	
**Medications**			
ACEI or ARBn (%)	11 (20.0)	12 (27.3)	0.39
Beta-blockers n (%)	20 (36.4)	17 (38.6)	0.82
Digitalis n (%)	3 (5.5)	0 (0)	n/a
Diuretics n (%)	13 (23.6)	13 (29.5)	0.51
Statins n (%)	13 (23.6)	7 (15.9)	0.34
**Examinations**			
EF < 40% n (%)	6 (10.9)	2 (4.5)	0.25

Note: ACEI: ACE- inhibitors; ARB; Angiotension receptor blockers; EF: Ejection fraction; IHD; Ischaemic heart disease; NYHA: New York Heart Association functional class; *p* values indicate the Chi-square test was used for discrete variables.

**Table 2:** Metabolites that showed significantly lower levels in plasma of men receiving a daily addition of SeQ_10_ for 18 months as compared with the controls, the placebo-treated men.

Metabolite	*p*-Value	VIP (OPLS-DA)
***Decreased in SeQ_10_ group***		
Alanine	0.001	1.79
Alanine-3-cyano	0.016	1.62
Arachidonic acid	NS	1.30
Aspartic acid	0.017	1.65
Butyric acid, 2-amino	NS	1.16
Cysteine	0.048	1.05
Fucose	NS	1.21
Glutamic acid	0.022	1.60
Glycine	0.010	1.50
Isoleucine	0.031	1.49
Leucine	NS	1.45
Methionine	0.030	1.51
Myo-inositol	0.022	1.42
Ornithine-1,5-lactam	0.013	1.29
Phenylalanine	NS	1.25
Proline-4-hydroxy	0.026	1.39
Salicylic acid	NS	1.34
Serine	0.032	1.44
Threonine	0.049	1.36

Note: No metabolite showed significantly higher levels in SeQ_10_ men; Note: VIP: Variables’ Importance to the Projection. Note: T-tests were used in the statistical evaluation. Note: NS: Not significant.

**Table 3:** Compounds that were found significant according to T-test in at least one batch when SeQ_10_ and controls at 18 months were compared, with the corresponding *p*-value and VIP value from this batch.

Compound	*p* Value (95% Confidence Level)	VIP
***Decreased in the SeQ_10_ group***		
Alanine	0.026	1.94
Glycine	0.025	1.95
Isoleucine	0.016	1.93
Leucine	0.031	1.60
Lysine	0.025	1.37
Tryptophan	0.025	0.95
*Increased*		
1-monohexadecanoylglycerol	0.004	1.79
Aspartic acid	0.007	2.19
Hexadecanoic acid	0.013	1.71
Hexadecenoic acid	0.039	1.63
Lauric acid	0.008	1.51
Myristic acid	0.026	1.44
Oleic acid	0.033	1.72

Note: *p*-values obtained from *t*-tests. Note: VIP values were obtained from the OPLS-DA model.

**Table 4:** Compounds that were found significant according to *t*-test in at least one batch when SeQ_10_ and controls for men at 18 months were compared, with the corresponding *p*-value and VIP value from this batch.

Compound	*p* Value (95% Confidence Level)	VIP
***Decreased in the SeQ_10_ group***		
Arabinose	0.013	1.83
Ribose	0.014	1.76
Sucrose	0.034	1.83
Xylitol	0.025	1.41
*Increased*		
Fructose	0.022	1.96
1,5-anhydroglucitol	0.016	1.93

Note: *p* values obtained from *t*-tests; VIP values were obtained from the OPLS-DA model.

**Table 5:** Metabolites identified in the main study and validation studies 1 and 2 together with the combined loading vectors from these studies.

Compound	Main Study	Validation 1	Validation 2
1,5-anhydro-d-glucitol	N	↑	↑
1-dodecanoyl-sn-glycero-3-phosphocholine	N	N	↑
1-Monohexadecanoylglycerol	N	↑	↑
1-Palmitoyl-sn-glycero-3-phosphocholine	N	↓	N
2-aminobutyric acid	N	↓	↓
3-hydroxybutyric acid	N	↑	↑
Alanine	↓	↓	↓
Alanine, 3-cyano	↓	N	N
Allothreonine	N	↓	N
Alpha-ketoglutarate	↓	N	N
Aminomalonic acid	N	N	↓
Arabinose	↓	↓	↓
Arabitol/ribitol	↓	N	N
Arachidonic acid	↓	N	N
Arginine	↓	↓	↓
Asparagine, DL-	↓	↓	↓
Aspartic acid, DL	↓	↓	↑
Benzyl alcohol	↓	N	N
Beta-Alanine	↓	N	N
Butanoic acid, 2,4-dihydroxy-	↑	N	N
Butyric acid, 2-amino	↓	N	N
Campesterol	↑	↑	↑
Cholesterol	↑	N	N
Citric acid	↓	↑	↑
Creatinine	↓	↓	↑
Cysteine	↓	↓	↓
Cystine	↓	↑	↓
Docosanoic acid	↑	↑	N
Docosahexaenoic acid, 4,7,10,13,16,19-(Z,Z,Z,Z,Z,Z)	↓	N	↓
Eicosanoic acid, n-	↓	N	N
Eicosapentaenoic acid	↓	N	N
Ethanolamine	↓	↑	↑
Fructose	↑	↑	↑
Fucose	↓	N	N
Glucose	↑	N	N
Glucose, 1,6-anhydro, beta	↓	↓	N
Glutamic acid	↓	↓	↓
Glutamine	↓	↓	↑
Glyceric acid	↓	↓	↓
Glycerol	↑	N	N
Glycerol-2-phosphate	↓	N	N
Glycerol-3-phosphate	↑	↓	↓
Glycine	↓	↓	↑
Glycolic acid	N	N	↑
Heptadecanoic acid, n-	↑	↓	N
Heptanoic acid	↑	N	N
Hexadecanoic acid	↑	↑	↑
Hexadecenoic acid	↑	↑	↑
Hippuric acid	↓	N	N
Histidine	↓	↓	↓
Indole-3-acetic acid	↓	N	N
Inosine	↑	N	N
Inositol, myo	↓	↑	↓
Inositol-1-phosphate	↑	↓	↑
Isoleucine	↓	↓	↓
Itaconic acid	↓	N	N
Lactic acid,	↓	↓	↑
Lauric acid (dodedecanoic acid)	↑	↑	↑
Leucine	↓	↓	↓
Linoleic acid	↑	↑	↑
Lysine	↓	↓	↓
Malic acid	↓	↓	↑
Malonic acid, 2-amino	↑	N	N
Maltose	↓	↓	↑
Mannitol	N	N	↓
Mannose	↑	N	N
Methionine	↓	N	N
Monomethylphosphate	↑	↑	↑
Myristic acid	↑	↑	↑
Nonanoic acid,n	↓	↑	↓
Oleic acid	↑	↑	↑
Ornithine	↓	↓	↓
Ornithine-1,5-lactam	↓	N	N
Oxalic acid	N	N	↓
Phenylalanine	↓	↓	↓
Phosphoric acid	↑	N	N
Pipecolic acid	↓	N	N
Proline	↓	↓	↓
Proline, 4-hydroxy	↓	N	N
Protocatechuic acid	↓	N	N
Pseudouridine	N	↓	↑
Putrescine	↓	N	N
Pyridine, 3-hydroxy	↓	N	N
Pyroglutamic acid	↓	↓	↑
Pyruvic acid	↑	↓	↓
Ribose	↓	↓	↓
Salicylic acid	↓	↓	↓
Serine	↓	↓	↓
Sitosterol	↑	↓	N
Sorbitol	↑	N	N
Stearic acid	↑	↑	↑
Succinic acid	↑	N	↑
Sucrose	↑	↑	↑
Taurine	↓	↓	↓
Threitol	↑	N	N
Threonic acid	↓	↑	↓
Threonine	↓	↓	↓
Tocopherol, alpha-	↑	↑	↓
Tocopherol, gamma-	↓	↓	↑
Tryptophan	↓	↓	↓
Tyrosine	↓	↓	↓
Urea	↓	N	↓
Uric acid	↓	↓	↑
Valine	↓	↓	↓
Xylitol	↑	↓	↓
Xylose	↓	↓	↑
Xylulose	↓	N	↑

Note: Arrows pointing down are metabolites with lower levels in the SeQ_10_-treated men compared with the placebo group, and arrows pointing up are those metabolites that showed higher levels in the SeQ_10_ treated men. “N” stands for no change as a result of the intervention.
